# The pathogenesis of renal injury and treatment in light chain deposition disease

**DOI:** 10.1186/s12967-019-02147-4

**Published:** 2019-11-25

**Authors:** Qi Wang, Fang Jiang, Gaosi Xu

**Affiliations:** 1 Department of Nephrology, The Second Affiliated Hospital of Nanchang University, No. 1, Minde Road, Donghu District, Nanchang, Zip Code: 330006 People’s Republic of China; 2 Medical Center of the Graduate School, Nanchang University, Nanchang, China; 3 Department of Nephrology, People’s Hospital of Xinyu City, No. 369, Xinxin North Avenue, High-tech District, Xinyu, People’s Republic of China

**Keywords:** Light chain deposition disease, Renal injury, Biopsy, Chemotherapy, Treatment

## Abstract

Light chain deposition disease (LCDD) is a rare clinical disorder. The deposition of light chain immunoglobulins mainly affects the kidneys, which have different characteristics than other tissues. To date, the therapeutic approach for the treatment of LCDD has no evidence-based consensus, and clinical experience of reported cases guides current disease management strategies. The present systematic review investigates and summarizes the pathological mechanisms of renal injury and the subsequent treatments for LCDD.

## Background

Monoclonal immunoglobulin deposition disease (MIDD) is a multi-system disease characterized by the deposition of monoclonal Ig molecules in various organs [1, 2]. Light chain deposition disease (LCDD) is the most common form of MIDD diagnosed, and it is a systemic disease. Many organs are affected by the deposition of monotype immunoglobulin light chain (LCs), but the kidneys are always affected [3]. Primary plasma cell abnormalities or other lymphoproliferative diseases are usually associated with the pathology of LCDD. However, morphological renal lesions (i.e., the presence of nodular sclerosis and the distribution of deposits) do not seem to correlate with patient survival in LCDD [4]. Because free light chains (FLCs) are rapidly cleared from the serum and are largely filtered by the kidneys, this organ is a prominent target for LC deposition and is often damaged. Clinically, LCDD is characterized by prominent mesangial nodules, a thickening of the peripheral basement membrane, and the extensive deposition of monoclonal LCs. Renal involvement in LCDD presents as renal lesions, hypertension, microhematuria, proteinuria and, more rarely, renal tubular acidosis. Extrarenal lesions are present in 35% of patients and can cause clinical symptoms, and extrarenal LC deposition has a clear, independent effect on patient survival [4].

LCDD is relatively rare and it is frequently misdiagnosed as a protein disease. Up to 50% of patients are diagnosed with LCDD secondary to multiple myeloma or other lymphoproliferative diseases. The diagnosis of LCDD can be made with a kidney biopsy. The characteristic morphological findings in LCDD are nodular glomerulosclerosis and nonfibrillar electron-dense deposits on the glomerular or tubular basement membrane, as seen with electron microscopy (EM). To date, there is no unified standard for the treatment of primary LCDD.

## The pathological manifestations of LCDD

### Light microscopy (LM)

In total, 30–100% of LCDD patients in the United States and France are characterized by nodular glomerulosclerosis [5, 6]. Under LM, LCDD shows the glomeruli with nodular mesangial expansion, a thickening and wrinkling of the glomerular basement membrane (GBM), and glomerular peripheral capillary walls with focal irregular thickening. Periodic acid–Schiff (PAS) staining is positive in LCs. The deposits in LCDD are neither fibrillar nor stained by Congo red. Milder forms of LCDD are characterized by moderately thickened basement membrane and an increased mesangial matrix and cells. Glomerular lesions require ultrastructural examination when not detected by LM.

### Immunofluorescence (IF)

IF examination of the kidney has been a key step in the diagnosis of LCDD. Along tubular basement membranes, monotypic LCs (mostly κ) are found in kidney biopsy specimens. The diagnosis of LCDD requires this evaluation. IF can reveal staining of LCs, either kappa or lambda, along the mesangial nodules, peritubular regions, vessels, interstitium, and GBM. However, the staining of IgG, IgA, IgM, and C3 is negative.

### Electron microscopy (EM)

Electron-dense, nonfibrillar, amorphous deposits in the GBM and tubular basement membranes are seen under EM [6]. Subendothelial linear punctate to powdery deposits are distributed in the capillary walls, while podocyte foot processes are largely preserved. EM depicts these deposits as dark granular electron densities. Under EM, dense granular deposits should be present in the mesangial area and subendothelial space without fibrillar structures. However, 8% of patients with LCDD have 8–20 nm fibrillar structures [7]. The fibrillar structure gradually replaces the normal matrix, leading to the destruction of the glomerular architecture [8–10]. The morphological, immunofluorescence staining and ultrastructural characteristics are summarized in Table 1.

**Table 1:** Results of renal histopathological examination in patients with LCDD

	Light microscopy (LM)	Immuno-fluorescence (IF)	Electron microscopy (EM)
Glomerular	Mild to moderate nodular mesangial expansion	Linear, either kappa or lambda, LC restricted staining of glomerular, negative for IgG, IgA, IgM, and C3	Dark powdery electron dense deposits along the inner aspects of glomerular basement membranes, or nodular glomerulosclerosis with abundant powdery to vaguely organized electron dense deposits in the expanded and condensed mesangium
Tubular	Thickening and wrinkling of the tubular basement membranes	Monotypic LC (mostly κ) fixation along tubular basement membranes, negative for IgG, IgA, IgM, and C3	Linear punctate to powdery deposits along tubular basement membranes
The small arterioles	Focal irregular thickening of the capillary walls	Strong LC staining along the peritubular capillary, negative for IgG, IgA, IgM, and C3	Diffuse subendothelial linear punctate to powdery deposits with mostly preserved the capillary walls

*LC* light chain

## The pathogenesis of renal injury in LCDD

**Fig. 1:**
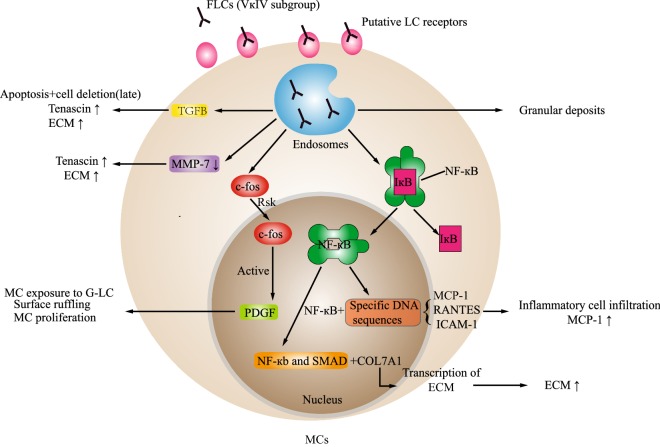
The interaction between light chain deposition disease (LCDD) free light chain (FLCs) and mesangial cells (MCs): FLCs enter MCs through a putative receptor. LCDD FLCs are processed in endosomes. The processed FLCs are deposited on the membrane of mesangial cells as granular deposits. Meanwhile, transforming growth factor (TGF)-β production is increased and matrix metalloproteinase (MMP)-7 is decreased, resulting in an increase in ECM and tenascin. Furthermore, TGF-β leads to apoptosis and the late deletion of cells. Nuclear factor kappa-light-chain-enhancer of activated B cells (NF-κB), as a dimer of P50 and p65 subunits, usually exists in the cytoplasm of MCs, binding to its inhibitor protein, IκB. When LCs stimulate MCs, IκB is released from the dimer, resulting in NF-κB migration to the nucleus. NF-κB binds to specific DNA (MCP-1, RANTES, ICAM-1), leading to inflammatory cell infiltration and an increase MCP-1. The functional interaction between NF-κB and SMAD leads to the activation of COL7A1 expression, resulting in an increase in ECM. Ribosomal S6 kinase (RSK) phosphorylates c-fos. Then the activation of c-fos results in the transcription of PDGF-β. PDGF induces MCs to be exposed to monoclonal LC, and cell surface wrinkling increases the cell surface area and promotes MC early proliferation. *LCDD* Light chain deposition disease, *FLC* Free light chain, *ECM* Extracellular matrix, *TGF-β* Transforming growth factor-β, *MMP-7* Matrix metalloproteinases-7, *RSK* Ribosomal S6 kinase, *NF-κB* Nuclear factor kappa-light-chain-enhancer of activated B cells, *PDGF* Platelet-derived growth factor, *MCP* Monocyte chemoattractant protein, *RANTES* Regulated upon activation normal T-expressed and secreted, *ICAM-1* Intercellular adhesion molecule-1

Glomerular-filtered FLCs are reabsorbed in the mesangium or proximal tubules. Mesangial cells (MCs) secrete extracellular matrix (ECM), mediators and enzymes such as matrix metalloproteinases (MMPs) to support and maintain the glomerulus [11–18]. The increasing deposition of ECM proteins and monotypic LCs results in mesangial nodularity within the glomerulus. MCs are critical in the pathogenesis of glomerulosclerosis. Figure 1 summarizes the interactions of MCs with glomerulopathic FLCs.

FLCs bind to putative receptors residing in caveolae present on the plasma membrane of MCs to initiate intracellular signalling [19, 20]. This signalling leads to the overexpression of the receptor [20]. The majority of monoclonal LCs in LCDD are κ, specifically the VκIV subgroup [2, 21–23]. The complementarity-determining region (CDR) of LCDD-associated FLCs has unusual hydrophobic amino acids (AA) substitutions [24], and κ-LCs in LCDD have an exposed b-edge that is part of the antigen binding site in the CDR2 loop, whereas λ-LCs do not [25]. This exposed edge leads to spontaneous aggregation of the k-LCs into oligomers, which may eventually form granular deposits [25]. The VκIV subgroup, which is frequently overrepresented in LCDD, has a particularly long CDR1 loop [26]. The CDR1 loop may promote conformational changes or the aggregation of the FLCs through its multiple hydrophobic residues. LCDD FLCs inhibit the release of MMP-7 from MCs [27]. MCs in LCDD show a significant decrease in the expression of MMP-7, which degrades tenascin-C [28], resulting in increased ECM.

Ribosomal S6 kinase (RSK) can phosphorylate a variety of transcription factors, including c-fos, promoting nuclear signal transduction [29]. C-fos acts via platelet-derived growth factor (PDGF)-β to further increase interactions with FLCs [19]. Nuclear factor kappa-light-chain-enhancer of activated B cells (NF-κB) and c-fos are induced to migrate to the nucleus by LCDD-associated FLCs [19]. The activation of c-fos results in the transcription of PDGF-β. PDGF-β mediates effects on MCs when exposed to glomerular LCs [30]. PDGF induces human fibroblast cell membrane wrinkling [31]. Previous studies have shown that the activation of the transcription factor NF-κB plays an important role in interleukin-1 (IL-1)-induced monocyte chemoattractant protein-1 (MCP-1) expression [29, 32]. Rovin et al. [33] proposed that phosphotyrosine kinase signalling mechanism could stimulate NF-κB, but this is not generally accepted [34]. NF-κB translocates into the nucleus and binds to specific DNA sequences on NF-κB response genes, such as MCP-1, regulated upon activation normal T-expressed and secreted (RANTES), and ICAM-1, resulting in enhanced transcription and generation [19]. Kon and colleagues have shown a functional interaction between NF-κb and SMAD, two early-intermediate transcription factors, to activate COL7A1 expression, an ECM-related gene [35].

When MCs are exposed to FLCs in LCDD, transforming growth factor (TGF)-β production is increased. Then, TGF-β inhibits mesangial proliferation and increases ECM secretion, including tenascin [36].

Cast formation can be seen in as many as one-third of LCDD cases [4]. Tubulointerstitial inflammation and fibrosis are the main features of cast formation, with hard and often fractured protein deposits in distal renal tubules (casts), composed of uromodulin and FLCs [37, 38]. Moreover, glomerular capillary walls have deposits of FLCs.

## Current treatments and outlook for novel therapies

The natural course of LCDD is associated with a very poor prognosis, and serum creatinine levels are higher than 1.2 mg/dL (average 3.9 mg/dL) at the diagnosis of LCDD in 97% of patients at the Mayo Clinic; 39% of patients developed end-stage renal failure over 34 months of observation, and 32% of patients died at a mean observation duration of 18 months [21]. The combination of multiple myeloma (RR = 2.75) and extrarenal deposition (RR = 2.24) are prognostic risk factors [39].

Currently, first-line combination chemotherapy and/or autologous stem cell transplantation (ASCT) are commonly used treatments [40–42]. However, thalidomide, dexamethasone, bortezomib, lenalidomide and other immunomodulators have not been widely recognized in LCDD, and further studies, especially prospective studies, are needed [2]. Drugs used to treat multiple myeloma are recommended when LCDD patients also have multiple myeloma. In patients with LCDD that is not accompanied by multiple myeloma, haematopoietic stem cell transplantation (HSCT) and chemotherapy with thalidomide, dexamethasone, bortezomib, lenalidomide, and alkylating drugs are recommended [2]. The proteasome inhibitor bortezomib, which directly interferes with and inhibits NF-κB, is a promising drug for reducing the formation of glomerular nodular lesions [43]. Peripheral neuropathy may be induced by both thalidomide and bortezomib because peripheral neuropathy symptoms improved after the end of treatment. It is very important to use adequate drugs to reduce the levels of free light chain. Apart from age, the degree of renal insufficiency at presentation, extrarenal LC deposition and underlying haematopoietic disorders affect patient outcomes [4].

Characteristics and responses to therapy in the included studies are shown in Table 2. A case report was published of a patient with LCDD who responded to MEVP (melphalan + cyclophosphamide + vincristine + prednisolone) chemotherapy, with no nodular glomerular lesions 7 years after MEVP treatment [44]. A complete haematological response, marked with a reduction in proteinuria, and improved renal function were observed in another patient with idiopathic LCDD that was treated with vincristine + dexamethasone (VD) [45].

**Table 2:** Included studies and response to therapy

Therapy	Study	Total	With MM	LC type		Age	Creatinine clearance (mL/min)		Median creatinine mg/dL		24 h urinary protein excretion	CR (%)	LC deposits	Surviving patients (%)	Median follow up (months)
				κ	λ		Pre	Post^a^	Pre	Post^a^					
MEVP	Komatsuda [44]	1	1	1	0	64	44	73	2.0	1.0	Decease to less than 0.1	100	0	100	70
BD	Kastritis [47]	4	0	3	1	57.85 (range 46–67)	–	–	2.9	1.9	Reduction 89.5%	50	2/4	100	11.3 (range 2–16)
HDM + ASCT	Kastritis [47]	3	0	3	0	51.3 (range 46–56)	–	–	–	–	Only trace proteinuria	100	–	100	14.3 (range 12–16)
After BD	González –López [23]	1	0	1	0	63	17	50	5.2	2.31	–	100	0	100	54
	Tovar [24]	3	0	1	2	49 (range 46–56)	–	–	2.14	1.27	Reduction 84%	67	0	100	28.7 (range 12–40)
ASCT after CBD	Smita [48]	1	0	1	0	33	–	–	4.9	1.60	–	100	0	100	12
and VLD	Weichman [40]	5	0	3	2	44.5 (range 36–51)	–	–	55	2 patients HD/PD	Reduction 75.3%	80	0	100	12 (range 4–29)
HDM + ASCT	Lorenz [46]	6	3	5	1	43.5 (33–61)	–	–	2.4	1 patient HD	Reduction 92%	100	–	83.3	31.7
	Pozzi [4]	5	4	–	–	58 ± 14.2	–	–	–	1 patient uremia	–	–	–	100	27.5

Complete hematologic response (CR) is defined as the complete disappearance of monoclonal Ig protein in serum and urine, the normalization of light chain ratio without serum, and the < 5% plasma cells without clonal advantage of k or l subtype demonstrated by bone marrow biopsy

*MM* Multiple myeloma, *LC* light chain, *MEVP* melphalan + cyclophos + phamide + vincristine + prednisolone, *BD* bortezomib + dexamethasone, *HDM* high-does melphalan, *ASCT* autologous stem cell transplantation, *CBD* cyclophosphamide + bortezomib + dexamethasone, *VLD* bortezomib + lenalidomide + dexamethasone

^a^Post-treatment values are from the last follow-up visits

ASCT is still an effective treatment regimen for LCDD that achieves long-term haematological responses [23, 40, 46]. Lorenz et al. [46] described the outcomes of 6 patients who underwent ASCT, and kidney function and renal response were significantly improved in 4 of the 6 patients with LCDD [46].

After intensive chemotherapy, ASCT can completely alleviate the dysplasia of plasma cells in LCDD [41]. One patient developed uraemia after a median follow-up of 44 months, but none of the 5 patients who had been treated with chemotherapy + ASCT died [4]. Bortezomib-based induction, followed by a combination of HDM (high-dose melphalan) and ASCT, has been used in several studies. In 2009, bortezomib combined with dexamethasone was used to treat 4 patients with LCDD [47]. Complete haematological responses were achieved in two patients, with serum-free LCs reduced by > 50% and improved renal function in another two patients. Three patients who underwent HDM + ASCT had complete haematological responses and only microalbuminuria. Non-HDM patients had proteinuria recurrence after 2 months but no haematological recurrence. In 2012, Tovar et al. [24] treated 3 patients with LCDD with bortezomib induction followed by HDM-conditioned ASCT, and 2 of the 3 patients showed rapid and significant improvement in renal function, but the remaining patient still had proteinuria residue. These reports show that the combination of bortezomib and dexamethasone followed by HDM-conditioned ASCT is a well-tolerated and effective treatment strategy for LCDD patients. Therefore, ASCT should be used as an intensifying therapy to achieve a response to chemotherapy induction that is tolerable. In addition, a pregnant patient with LCDD who responded to chemotherapy and ASCT remained in clinical remission with normal serum electrophoresis results at her 1-year follow-up [48].

Dialysis is worth performing in uremic LCDD patients. Uraemia per se does not adversely impact survival, and renal replacement therapy (RRT) is beneficial for patients with LCDD who have achieved uraemic status. Two types of dialysis (peritoneal dialysis and haemodialysis) have similar chances of survival [4].

For some LCDD patients, renal transplantation (RTX) is a good choice after ASCT [49, 50]. RTX should not be considered if there is persistent disease or no previous treatment to control FLC production. Otherwise, the transplanted kidney will suffer injuries similar to the patient’s original kidney [23, 51, 52]. It has been reported that the recurrence rate of LCDD is more than 50% within 4 years after kidney transplantation and is often associated with transplant failure [53, 54]. Leung et al. described that LCDD recurred in 5 of 7 kidney transplants [54]. The earliest recurrence of LCDD after RTX was 2.9 months, and the median recurrence time was 33.3 months. Moreover, there was a case report of a triple approach that combined ASCT, RRT, and nonmyeloablative ASCT in a young woman with κ-LCDD. She was in complete remission for over 9 years, and no immunosuppressive treatment was required [55].

## Conclusions

The present article summarizes that immunofluorescence examination of the kidney is necessary for diagnosis and that MCs are critical in the pathogenesis of glomerulosclerosis. Renal transplantation is a good choice when free light chains production is under control.

## Data Availability

All data generated and analysed during this study are included in this published article.

## Abbreviations

LCDD: light chain deposition disease
LCs: light chains
FLCs: free light chains
LM: light microscopy
GBM: glomerular basement membrane
PAS: periodic acid–Schiff
IF: immunofluorescence
EM: electron microscopy
ECM: extracellular matrix
MMPs: matrix metalloproteinases
MCs: mesangial cells
CDR: complementarity-determining region
AA: amino acids
NF-κB: nuclear factor kappa-light-chain-enhancer of activated B cells
PDGF-β: platelet-derived growth factor
IL-1: interleukin-1
MCP-1: monocyte chemoattractant protein-1
RANTES: regulated upon activation normal T-expressed and secreted
ICAM-1: intercellular adhesion molecule1
TGF-β: transforming growth factor-β
ASCT: autologous stem cell transplantation
HDM: high-dose melphalan
RTX: renal transplantation
PD: peritoneal dialysis
HD: hemodialysis
